# Ivermectin in Acute Renal Injury Induced by Ischemia-Reperfusion: An Experimental Study in Wistar Rats

**DOI:** 10.1590/0034-7167-2025-0104

**Published:** 2026-07-06

**Authors:** Raphael Cuencas Budini, Dulce Aparecida Barbosa, Iris Gabriela Piazentin de Oliveira, Ricardo dos Santos Silva, Adriana Almeida de Sousa, Milene Subtil Ormanji Thibes, Cassiane Dezoti da Fonseca

**Affiliations:** I Universidade Federal de São Paulo. São Paulo, São Paulo, Brazil; II Universidade Federal de Sergipe. São Cristóvão, Sergipe, Brazil

**Keywords:** Reperfusion Injury, Acute Kidney Injury, Ivermectin, SARS-CoV-2, Oxidative Stress., Daño por Reperfusión, Lesión Renal Aguda, Ivermectina, SARS-CoV-2, Estrés Oxidativo.

## Abstract

**Objectives:**

to assess the effect of ivermectin in an experimental model of ischemia-reperfusion as a risk factor.

**Methods:**

twenty adult male Wistar rats were randomized into the following groups: Sham: surgical simulation without clamping of the renal pedicles; ischemia: clamping of the renal pedicles for 30 minutes; Sham + ivermectin: 10 mg/kg of ivermectin, orally, once daily for five days; and Ischemia + ivermectin. Physiological parameters such as body weight, water and food intake, renal function (creatinine clearance), and oxidative profile (urinary peroxides and thiobarbituric acid reactive substances) were assessed.

**Results:**

an increase in kidney-to-body weight ratio, serum creatinine, and oxidative stress was observed in animals treated with ivermectin (p<0.05).

**Conclusions:**

the use of ivermectin, associated with the ischemia-reperfusion risk factor, demonstrated nephrotoxic action in Wistar rats, evidenced by impaired renal function and increased oxidative stress.

## RESUMEN

**Objetivos:**

evaluar el efecto de la ivermectina en un modelo experimental de factor de riesgo de isquemia-reperfusión.

**Métodos:**

veinte ratas Wistar macho adultas se asignaron aleatoriamente a los siguientes grupos: Sham: cirugía simulada sin pinzamiento del pedículo renal; isquemia: pinzamiento del pedículo renal durante 30 minutos; Sham + ivermectina: 10 mg/kg de ivermectina por vía oral una vez al día durante cinco días; e isquemia + ivermectina. Se evaluaron parámetros fisiológicos como el peso, la ingesta de agua y alimento, la función renal (aclaramiento de creatinina) y el perfil oxidativo (peróxidos urinarios y sustancias reactivas al ácido tiobarbitúrico).

**Resultados:**

se observó un aumento en la relación peso/riñón, la creatinina sérica y el estrés oxidativo en los animales que recibieron ivermectina (p<0,05). La isquemia-reperfusión asociada a la ivermectina empeoró aún más estos parámetros (p<0,05).

**Conclusiones:**

el uso de ivermectina, asociado al factor de riesgo de isquemia-reperfusión, demostró efectos nefrotóxicos en ratas Wistar, evidenciados por una función renal deteriorada y un aumento del estrés oxidativo.

## INTRODUCTION

Acute kidney injury (AKI) is defined by an absolute increase of 0.3 mg/dL (26.5 µmol/L) or a 1.5-fold rise from baseline serum creatinine, and/or by urine output lower than 0.5 mL/kg/hour for a period between six and twelve hours^(1)^.

The kidney is a particularly vulnerable organ and, under clinical conditions such as hypovolemia, severe dehydration, and heart failure, it may rapidly progress to dysfunction. In such cases, a major pathophysiological mechanism resulting from severe hypoxia is ischemia-reperfusion, which involves hemodynamic and cellular alterations^(2,3)^. During the ischemic phase, anaerobic metabolism predominates, leading to lactic acid accumulation and reduced synthesis of endogenous antioxidants. Upon reperfusion, aerobic metabolism is restored, resulting in an abrupt increase in reactive oxygen species (ROS) production. This redox imbalance causes tissue injury through oxidative stress, triggering inflammation, necrosis, and apoptosis^(4-6)^. In this context, the use of nephrotoxic drugs must be carefully assessed, considering the risk-benefit ratio.

In recent years, in the face of pandemics and epidemics of neglected diseases, particularly in developing countries, the need to explore drugs with potential therapeutic applications has increased^(7,8)^. Among them, ivermectin has gained attention. Initially developed as an antiparasitic agent, it has demonstrated, in *in vitro* studies, the ability to inhibit nuclear import of host proteins, including the translocation of dengue virus non-structural protein 5^(9,10)^. Furthermore, its use in patients with COVID-19 was associated with reduced mortality, shorter hospital stays, and faster viral clearance^(11,12)^. However, dose-dependent adverse effects such as headache, fever, and blurred vision have also been reported^(13-15)^.

Despite these findings, little is known about the relationship between ivermectin and kidney injury. An experimental animal study showed that its administration induced expression of the P2X4 receptor, associated with tubular necrosis, neutrophil infiltration, and apoptosis^(16)^.

Considering that ivermectin is not currently recommended for the treatment or prophylaxis of diseases such as dengue and COVID-19, this experimental study in Wistar rats may contribute to understanding the pathophysiological mechanisms involved in the interaction between ivermectin and ischemia-reperfusion-induced AKI^(14,16)^.

### Study relevance

This article is the product of a final course project^(17)^, which is included in the institutional repository https://repositorio.unifesp.br/handle/11600/69385.

## OBJECTIVES

To assess the effect of ivermectin in an experimental model of Wistar rats with ischemia-reperfusion risk factor.

## METHODS

### Ethical aspects

The study was conducted in accordance with national and international ethical guidelines^(18)^, and was approved by the *Universidade Federal de São Paulo* Ethics Committee on Animal Use.

### Study design, period, and location

This was a quantitative experimental study using an animal model, guided by the ARRIVE guidelines, conducted at the *Escola Paulista de Enfermagem, Universidade Federal de São Paulo* (UNIFESP) in partnership with the Multi-User Animal Facility, located on the 8^th^ floor of research building I - UNIFESP, from August 2022 to August 2023.

### Sample

Twenty adult male Wistar rats, weighing between 250 and 290 g, were used. The sample size calculation was performed with a 5% sampling error and a 90% Confidence Interval. The resource equation method was used. This method is useful in complex experiments with multiple treatment groups. It involves the analysis of results using analysis of variance (ANOVA), considering the degrees of freedom in the analysis. The formula for calculating the degrees of freedom (E) is: E = N - G, where N is the total number of animals, and G is the total number of groups. The objective is to keep E between 10 and 20, ensuring an adequate sample size^(19)^. The animals in the different groups were kept with free access to water and food, and remained in thermal conditions with alternating day and night cycles.

### Study protocol

The animals were randomized into the following experimental groups: Sham (n=5): animals that were used for surgical procedure simulation, without clamping of the renal pedicles; ischemia (n=5): animals that were used for clamping of the renal pedicles for a period of 30 minutes; Sham + ivermectin (n=5): Sham animals that received 10 mg/kg of ivermectin, orally, once a day, for a period of five days; ischemia + ivermectin (n=5): ischemia animals that received 10 mg/kg of ivermectin, orally, once a day, for a period of five days^(16)^.

The ivermectin dosage was calculated based on the minimum risk of toxicity. The standard dose in humans is 0.2 mg/kg/day to 0.6 mg/kg/day for five days^(20)^. The use of the drug in Wistar rats was based on the calculation of the equivalent human dose, which is ten times higher. Therefore, the dose to be used in the animals would be between 20 mg/kg/day and 60 mg/kg/day for five days^(21)^. A sub-dose of 10 mg/kg/day for five days was chosen, given the animals’ pre-existing condition due to ischemia-reperfusion, as well as to mimic the dose used by Han *et al*.^(16)^.

The experimental protocol lasted eight days. On the first day, the animals were anesthetized with ketamine/xylazine (75 mg/kg, 10 mg/kg; Anasedan^®^, Vetbrands), both intraperitoneally, and subjected to ischemia-reperfusion (using non-traumatic vascular clamps) or a 30-minute sham surgery. After this period, the incision was closed, and the wound was cleaned and disinfected. The animals were monitored until anesthetic recovery and returned to the animal facility, where they remained at rest for 24 hours to recover from the surgical procedure, receiving tramadol (20 mg/kg) every eight hours. From the third to the seventh day, the animals received 10 mg/kg/day of ivermectin orally once a day. Afterwards, they were placed in metabolic cages for 24 hours to measure 24-hour urinary volume, water and food intake. On the eighth day, the animals were anesthetized with ketamine/xylazine (100 mg/kg/10 mg/kg; Anasedan^®^, Vetbrands) intraperitoneally, and a blood sample was collected by abdominal aorta puncture. During the procedure, the animals were kept on a heated surface at 38ºC to prevent hypothermia. The right kidney was removed and subsequently weighed to calculate the kidney weight to animal weight ratio. The urine sample was used for renal function studies and, subsequently, for oxidative stress studies.

At the end of the experiment, the animal was euthanized by physical exsanguination, according to the ethical guidelines for handling animals in research laboratories^(18)^.

### Renal function and biochemistry

To assess renal function, plasma and/or urinary levels of creatinine and urea were analyzed spectrophotometrically, according to standard procedures, using commercially available diagnostic kits (Labtest Diagnostic, MG, Brazil). Glomerular filtration rate was determined by creatinine clearance, calculated using the formula: creatinine clearance = urinary creatinine x urinary flow (24 hours) / serum creatinine^(22,23)^. Urea levels were determined using a colorimetric assay based on urease activity^(24)^. Urinary proteins were determined using a colorimetric method based on pyrogallol red-molybdate^(25)^. The results were expressed in mg/24 hours.

### Oxidative stress

Oxidative metabolites were assessed by measuring urinary peroxides and thiobarbituric acid reactive substances (TBARS). Urinary peroxide assessment was performed using the FOX-2 method, in which the use of ferro-xylenol orange oxidizes the Fe2+ ion, producing a bluish-purple colored complex (α= 4,3 x 104 M^-1^ cm^-1^)^(26-28)^. Urinary TBARS assessment allows the identification of end products of the lipid peroxidation cascade, which react in the presence of thiobarbituric acid in organic fluids (α= 1,56 x 105 M^-1^ cm^-1^)^(29)^.

### Acute kidney injury classification

The Kidney Disease: Improving Global Outcomes (KDIGO) organization established the main guideline for identifying/defining AKI and its stages. The classifications are presented in Chart 1 ^(1)^.

**Chart 1:** Kidney Disease: Improving Global Outcomes classification

KDIGO 1	Increase in serum creatinine ≥ 0.3 mg/dL (≥ 26,5 μmol/L) within 48 hours or an increase of 1.5 to 1.9 times the baseline value (occurring or presumed within seven days), or urine output < 0.5 mL/kg/hour for six to 12 hours.
KDIGO 2	Increase in serum creatinine of 2.0 to 2.9 times the baseline value or urine output less than 0.5 mL/kg/hour for ≥ 12 hours.
KDIGO 3	A three-fold increase in serum creatinine compared to baseline or serum creatinine ≥ 4.0 mg/dL (≥ 353.6 μmol/L) or initiation of renal replacement therapy (dialysis), or urine output less than 0.3 mL/kg/hour for ≥ 24 hours or anuria for ≥ 12 hours.

*KDIGO - Kidney Disease: Improving Global Outcomes.*

### Analysis of results and statistics

The assumptions of normality and homogeneity of variances were tested for all variables. Results were expressed as mean ± standard deviation when the variables adhered to the normality test. The comparison of variations between groups was verified using the One-Way ANOVA test, followed by Tukey’s multiple comparisons post-test using the Graph-Pad Prism version 8 statistical software for Windows^®^. Values of p<0.05 were considered significant.

## RESULTS

Table 1 shows that the animal weight in the Sham + ivermectin and ischemia + ivermectin groups varied significantly compared to the groups that did not receive ivermectin. Furthermore, the kidney weight/animal weight ratio showed an increase in the ischemia + ivermectin group compared to the others.

**Table 1:** Physiological parameters of the different groups, such as Sham, Sham + ivermectin, ischemia, and ischemia + ivermectin, São Paulo, São Paulo, Brazil, 2024

Groups	n	Animal weight (g)	Kidney weight (g)	Kidney weight/ animal weight	Feed intake (g)	Water intake (ml)
Sham	05	292 ± 15	1.24 ± 0.09	0.42 ± 0.03	32 ± 13	38 ± 5
Sham + ivermectin	05	243 ± 28^a^	1.20 ± 0.17	0.50 ± 0.09	34 ± 5	29 ± 10
Ischemia	05	249 ± 28	1.22 ± 0.36	0.48 ± 0.10	27 ± 16	30 ± 14
Ischemia + ivermectin	05	244 ± 20 ^a^	1.80 ± 0.53	0.75 ± 0.27^a^	25 ± 14	50 ± 23

The data were expressed as mean ± standard deviation;

a p<0.05 vs Sham.

**Figura 1:**
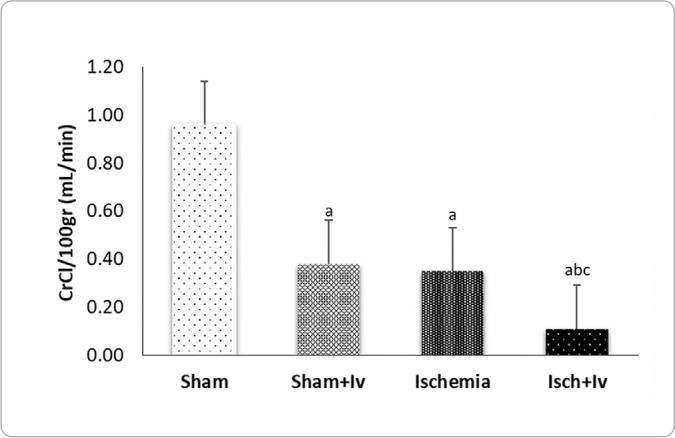
*Clearance* de Creatinina dos grupos Sham (0,96); Sham + IV (0,38); Isquemia (0,35); Isquemia + IV (0,11), São Paulo, São Paulo, Brasil, 2026

Table 2 shows that, despite the numerical increase in urinary volume in rats treated with ivermectin, and even more so in those subjected to the ischemia-reperfusion process, the results were not significant. In contrast, urinary creatinine showed significance in the ischemia + ivermectin group compared to the Sham group, as did plasma creatinine. Urea showed a significant increase in the group that combined ivermectin with ischemia-reperfusion. The analysis of creatinine clearance shown in Figure 1 demonstrated that the animals treated with ivermectin showed a decrease, which was even greater in the groups subjected to ischemia-reperfusion, especially in the ischemia + ivermectin group.

**Table 2:** Renal function of the different groups, such as Sham, Sham + ivermectin, ischemia, and ischemia + ivermectin, São Paulo, São Paulo, Brazil, 2024

Groups	n	24-hour urine volume (ml/min)	Urinary creatinine (mg/dL)	Plasma creatinine (mg/dL)	Proteinuria (mg/dL)	Urea (mg/dL)
Sham	05	0.011 ± 0.001	72.6 ± 14.5	0.30 ± 0.08	42.5 ± 5.3	39.9 ± 7.7
Sham + ivermectin	05	0.009 ± 0.002	57.9 ± 18.6	0.60 ± 0.06	44.7± 21.8	49.2 ± 18.4
Ischemia	05	0.011 ± 0.012	44.4 ± 14.1	0.69 ± 0.12	53.0 ± 14.9	61.8 ± 12.9
Ischemia + ivermectin	05	0.018 ± 0.014	27.9 ± 20.5^a^	1.26 ± 0.78^a^	42.4 ± 9.6	100.0 ± 23.2^abc^

The data were expressed as mean ± standard deviation;

a p< 0.05 vs Sham;

b p< 0.05 vs Sham + ivermectin;

c p< 0.05 vs ischemia.

*Os dados foram expressos em média ± desvio padrão. ap<0,05 vs Sham bp<0,05 vs Sham +IV cp<0,05 vs ISQ.*

**Figura 2:**
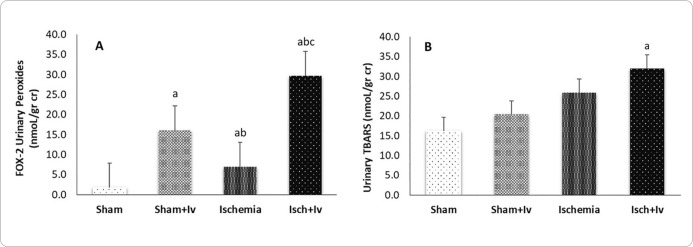
FOX e TBARS urinários. FOX (2A): Sham (1,74); Sham+IV (16,04); ISQ (6,98); Isq+IV (29,66). TBARS (2B): Sham (16,19); Sham+IV (20,40); ISQ (25,93); Isq+IV (32,01), São Paulo, São Paulo, Brasil, 2026

Figures 2A and 2B show the oxidative profile. It is observed that treatment with ivermectin was responsible for increasing FOX-2 levels, and when combined with the ischemia-reperfusion procedure, this increase was even greater. Furthermore, observation of urinary TBARS shows that the ischemia + ivermectin group presented a significant increase compared to the Sham group.

*Os dados foram expressos em média ± desvio padrão. ap<0,05 vs Sham bp<0,05 vs Sham +IV cp<0,05 vs ISQ.*

## DISCUSSION

This study investigated the relationship between ivermectin administration and the occurrence of renal injury in an experimental model of ischemia-reperfusion in Wistar rats over five days. A reduction in renal function associated with intense oxidative stress was observed in both the animals that received ivermectin alone and those subjected to the ischemia-reperfusion risk factor. These findings support the hypothesis of this study regarding the potential nephrotoxic effect of ivermectin.

The group of animals that received ivermectin showed significant weight variation during the experimental protocol. The inflammatory process caused by ivermectin ingestion and the ischemia-reperfusion surgical procedure, which results in energy consumption by the cells, were the main precursors for this weight reduction phenomenon. The release of pro-inflammatory cytokines directly or indirectly affects peripheral nerves, which stimulate areas of the brain inducing anorexia, in an attempt to prioritize energy for tissue and structure repair, mitigating the presence of pathogenic or toxic agents^(30,31)^.

Although the nephrotoxicity of ivermectin is not yet fully understood, a preclinical study demonstrated reduced renal function and damage to the renal parenchyma in animals treated for four days with 40 mg/kg of ivermectin-a dose equivalent to that administered in humans^(32,33)^. The present study showed elevated renal biomarkers after the use of 10 mg/kg of ivermectin for five days, supporting previous data and reinforcing the hypothesis of renal toxicity. This toxicity may be mediated by direct tubular damage, involving alterations in the polarity of cell membranes, with displacement of Na^+^/K^+^-ATPase pump proteins from the basolateral to the apical membrane, triggering cell apoptosis and DNA damage^(34-36)^.

In this context, an experimental study that administered 12 mg/kg of ivermectin to Wistar rats for five days demonstrated a reduction in Na^+^/K^+^-ATPase activity in the cerebral cortex, suggesting a possible correlation between the drug and cellular homeostasis dysregulation^(37)^.

Thus, in the renal parenchyma, an imbalance in the levels of water, sodium, potassium, and bicarbonate can be observed, in addition to an elevation of biomarkers such as urea and plasma creatinine, which can culminate in conditions of oliguria or polyuria^(4,5)^.

In the present study, the animals that were subjected to ischemia and received ivermectin showed an increase in urinary volume. This phenomenon can occur in models of ischemic AKI, since persistent hypoxia can cause vacuolization of the proximal tubule cells, resulting from the accumulation of giant lysosomes, which are also frequent in drug-induced AKI^(34,35)^. Thus, an unexpected increase in urine volume occurs through a mechanism known as osmotic nephrosis.

From a translational perspective, monitoring and assessing urine volume in patients at risk for AKI, whether in the community or in a specialized hospital care setting, can be mitigated by considering specific pathophysiological mechanisms, such as those described above. Therefore, not only is urine volume assessment fundamental, but also the analysis of renal biomarkers such as urea and creatinine, as well as clinical assessment through signs and symptoms such as edema and pulmonary congestion.

In an attempt to rationalize and intervene early in AKI clinical surveillance, KDIGO has been consolidating itself as a consensus among leading nephrologists worldwide^(1)^. This tool establishes criteria for AKI identification and stratification, guiding the multidisciplinary team in early diagnosis with the aim of promoting rapid intervention and facilitating patient recovery.

In this scenario, the present study demonstrated, through creatinine clearance and serum creatinine levels, that the preclinical experimental groups can be associated with the different stages proposed by KDIGO. Thus, in a translation from the bench to clinical practice, the animals in the control group could correspond to healthy patients (KDIGO 0); the groups that received ivermectin, to patients with isolated nephrotoxic AKI development (KDIGO 1); the group subjected to ischemia, to stage KDIGO 2; and the ischemia + ivermectin group, to stage KDIGO 3^(1,2)^.

In order to reinforce the hypothesis of ivermectin-induced acute renal injury, the oxidative metabolites assessed in this study demonstrated significant involvement of ROS, especially in the groups treated with ivermectin and those subjected to ischemic acute renal injury.

Analysis of urinary TBARS allows for lipid peroxidation monitoring, a process involving the degradation of lipid compounds by oxidizing agents’ action, resulting in changes in cell membrane permeability, the formation of toxic compounds, and consequently, cell death^(21)^. The analysis using the FOX-2 method allows for detecting peroxide levels, whose increase is associated with the intensification of ROS production resulting from oxidative stress^(21,22)^.

The data from the present study demonstrated a significant increase in urinary TBARS and FOX-2 levels, especially in the groups subjected to ischemia and ischemia associated with ivermectin administration. This finding can be explained primarily by the mechanisms involved in the ischemia-reperfusion process, since reperfusion promotes an abrupt increase in oxygen concentration, activating endothelial cells and recruiting inflammatory mediators and immune system cells, which intensifies oxidative stress^(38)^. Furthermore, the control group treated with ivermectin showed a statistically significant increase in FOX-2 levels, suggesting that ivermectin may act as a pro-oxidant compound, favoring peroxide formation.

A recent study with Wistar rats assessed ivermectin’s neurotoxicity, demonstrating increased levels of serum creatinine in animals that received a dose of 12 mg/kg of the drug for five days^(37)^. Furthermore, an increase in ROS and a reduction in the antioxidant enzyme catalase’s activity were observed. Another study on the chronic use of ivermectin, at a dose of 0.4 mg/kg, for four weeks, revealed a reduction in the activities of antioxidant enzymes, worsening of renal biomarkers, histological changes, and increased pro-apoptotic Bax protein expression^(39)^.

Persistent hypoxia in the renal cortex promotes the exacerbated production of ROS, which compromises mitochondrial oxidative phosphorylation, in addition to causing nitric oxide depletion, formation of damage-associated molecular patterns (DAMPs), activation of Toll-like receptors (TLRs), induction of autophagy, and microvascular dysfunction^(36,38)^.

In this context, DAMPs released by necrotic renal tubular cells activate TLRs present in renal cells, triggering a cascade that involves the NF-κB transcription factor activation. This process results in the nuclear translocation of NF-κB, promoting the expression of inflammatory cytokines such as tumor necrosis factor and interleukin-6, thus reinforcing the process of renal injury^(36,38)^.

Therefore, this study aimed to elucidate the pathophysiological mechanisms involved in ivermectin-induced renal toxicity by assessing renal function and redox imbalance in an experimental model in Wistar rats. The data presented may contribute to the construction of new paradigms regarding the use of this drug, especially in patients with risk factors.

### Study limitations

This study is limited by the absence of histological data that would allow us to demonstrate the renal morphological changes associated with the use of ivermectin. Furthermore, a dose-response analysis was not performed in experimental models with risk factors, despite the literature already describing the use of doses (19 mg/kg) considered safe in Wistar rats^(39)^.

### Contributions to nursing, health, or public policy

Nursing, as a key profession in providing comprehensive patient care, must be aware not only of the effects of administered medications but also of their interactions with potential risk factors. This knowledge is essential for adopting best practices in the care of patients with acute renal failure.

## CONCLUSIONS

Ivermectin is a potentially nephrotoxic drug, and its use should be cautious, as it was able to alter renal biomarkers, indicating worsening of renal function, and activate the oxidative process. Therefore, in this experimental study in Wistar rats, ischemia-reperfusion-induced AKI was characterized as a risk factor for ivermectin nephrotoxicity.

## Data Availability

The research data are available within the article.
