# Supplementary material for: Ivermectin in Acute Renal Injury Induced by Ischemia-Reperfusion: An Experimental Study in Wistar Rats
Source: Rev Bras Enferm. 2026 Jul 6;79:e20250104. doi: 10.1590/0034-7167-2025-0104 (PMC13336232; doi:10.1590/0034-7167-2025-0104)
Supplement: Translation

# Translation: Ivermectina na Injúria Renal Aguda por Isquemia-Reperfusão: Estudo Experimental em Ratos Wistar

**DOI:** 10.1590/0034-7167-2025-0104pt

**Authors:** Raphael Cuencas Budini, Dulce Aparecida Barbosa, Iris Gabriela Piazentin de Oliveira, Ricardo dos Santos Silva, Adriana Almeida de Sousa, Milene Subtil Ormanji Thibes, Cassiane Dezoti da Fonseca

**Affiliations:** I Universidade Federal de São Paulo. São Paulo, São Paulo, Brasil; II Universidade Federal de Sergipe. São Cristóvão, Sergipe, Brasil

**Keywords:** Traumatismo por Reperfusão, Injúria Renal Aguda, Ivermectina, SARS-CoV-2, Estresse Oxidativo.

## Abstract

**Objetivos:**

avaliar o efeito da ivermectina em modelo experimental de fator de risco isquemia-reperfusão.

**Métodos:**

utilizaram-se 20 ratos adultos machos da raça Wistar, randomizados nos seguintes grupos: Sham: simulação do ato cirúrgico, sem clampeamento dos pedículos renais; isquemia: clampeamento dos pedículos renais por 30 minutos; Sham + ivermectina: 10 mg/kg de ivermectina, via oral, uma vez ao dia, por cinco dias; e isquemia + ivermectina. Parâmetros fisiológicos, como peso, ingestão hídrica e de alimento, função renal (*clearance* de creatinina) e perfil oxidativo (peróxidos e substâncias reativas ao ácido tiobarbitúrico urinários), foram avaliados.

**Resultados:**

observou-se aumento da relação peso/rim, da creatinina sérica e do estresse oxidativo nos animais que receberam ivermectina (p<0,05). A isquemia-reperfusão associada à ivermectina favoreceu piora adicional desses parâmetros (p<0,05).

**Conclusões:**

o uso da ivermectina, associado ao fator de risco isquemia-reperfusão, demonstrou em ratos Wistar ação nefrotóxica, evidenciada pela piora da função renal e aumento do estresse oxidativo.

## INTRODUÇÃO

A injúria renal aguda (IRA) é definida pelo aumento absoluto de 0,3 mg/dL (26,5 µmol/L) ou pela elevação de 1,5 vezes em relação ao valor basal da creatinina sérica e/ou por débito urinário inferior a 0,5 mL/kg/hora por um período entre seis e 12 horas^(1)^.

O rim é um órgão particularmente vulnerável e, em condições clínicas como hipovolemia, desidratação grave e insuficiência cardíaca, pode evoluir rapidamente para disfunção. Nesses casos, um importante mecanismo fisiopatológico decorrente da hipóxia severa é a isquemia-reperfusão, que envolve alterações hemodinâmicas e celulares^(2,3)^. Durante a fase de isquemia, predomina o metabolismo anaeróbico, levando ao acúmulo de ácido lático e à redução da síntese de antioxidantes endógenos. Já na reperfusão, o metabolismo aeróbico é restabelecido, resultando em aumento abrupto na produção de espécies reativas de oxigênio (EROs). Esse desequilíbrio redox provoca lesão tecidual por estresse oxidativo, desencadeando inflamação, necrose e apoptose celular^(4-6)^. Nesse cenário, o uso de fármacos nefrotóxicos deve ser criteriosamente avaliado, considerando a relação risco-benefício.

Nos últimos anos, diante de pandemias e epidemias de doenças negligenciadas, sobretudo em países em desenvolvimento, cresceu a necessidade de explorar fármacos com potenciais terapêuticos^(7,8)^. Entre eles, destacou-se a ivermectina, inicialmente desenvolvida como agente antiparasitário, mas que demonstrou, em estudos *in vitro*, a capacidade de inibir a importação nuclear de proteínas do hospedeiro, incluindo a translocação da proteína não estrutural 5 do vírus da dengue^(9,10)^. Além disso, seu uso em pacientes com COVID-19 foi associado à redução da mortalidade, do tempo de internação e da progressão do *clearance* viral^(11,12)^. Entretanto, efeitos adversos dose-dependentes, como cefaleia, febre e visão turva, também foram relatados^(13-15)^.

Apesar dessas evidências, pouco se sabe sobre a relação entre ivermectina e injúria renal. Estudo experimental em modelo animal mostrou que sua administração induziu a expressão do receptor P2X4, associado à necrose tubular, infiltração de neutrófilos e apoptose^(16)^.

Considerando que o uso da ivermectina não é atualmente recomendado para tratamento ou profilaxia de doenças como dengue e COVID-19, este estudo experimental em ratos Wistar pode contribuir para o entendimento dos mecanismos fisiopatológicos envolvidos na interação entre ivermectina e injúria renal induzida por isquemia-reperfusão^(14,16)^.

### Relevância do estudo

Este artigo é produto de Trabalho de Conclusão de Curso^(17)^, o qual está inserido no repositório institucional https://repositorio.unifesp.br/handle/11600/69385.

## OBJETIVOS

Avaliar o efeito da ivermectina em modelo experimental de ratos Wistar com fator de risco por isquemia-reperfusão.

## MÉTODOS

### Aspectos éticos

O estudo foi conduzido de acordo com as diretrizes de ética nacionais e internacionais^(18)^, sendo aprovado pelo Comitê de Ética no Uso de Animais da Universidade Federal de São Paulo.

### Desenho, período e local do estudo

Estudo quantitativo experimental em modelo animal, norteado pela ferramenta ARRIVE, realizado na Escola Paulista de Enfermagem da Universidade Federal de São Paulo (UNIFESP) em parceria com o Biotério Multiusuário, localizado no 8º andar do prédio de pesquisa I - UNIFESP, durante o período de agosto de 2022 a agosto de 2023.

### Amostra

Foram utilizados 20 ratos da raça Wistar, machos e adultos, pesando entre 250 e 290 g. O cálculo amostral foi realizado com um erro amostral de 5% e Intervalo de Confiança de 90%. Foi utilizado o método da equação de recursos. Esse método é útil em experimentos complexos com múltiplos grupos de tratamento. Envolve a análise dos resultados por meio da análise de variância (ANOVA), considerando os graus de liberdade na análise. A fórmula para calcular os graus de liberdade (E) é: E=N-G, sendo que N é o número total de animais, e G, o número total de grupos. O objetivo é manter E entre 10 e 20, garantindo um tamanho amostral adequado^(19)^. Os animais dos diversos grupos foram mantidos com livre acesso à água e alimentos, e permaneceram em condições térmicas com ciclos alternados de dia e noite.

### Protocolo do estudo

Os animais foram randomizados nos seguintes grupos experimentais: Sham (n=05): animais que foram utilizados para simulação do ato cirúrgico, sem clampeamento dos pedículos renais; isquemia (n=5): animais que foram utilizados para o clampeamento dos pedículos renais por um período de 30 minutos; Sham + ivermectina (n=5): animais Sham que receberam 10 mg/kg de ivermectina, via oral, uma vez ao dia, durante um período de cinco dias; isquemia + ivermectina (n=5): animais isquemia que receberam 10 mg/kg de ivermectina, via oral, uma vez ao dia, durante um período de cinco dias ^(16)^.

A dose da ivermectina foi calculada base ada no risco mínimo de toxicidade. A dose padronizada em seres humanos é de 0,2 mg/kg/dia a 0,6 mg/kg/dia por cinco dias^(20)^. A utilização do fármaco em ratos Wistar foi subsidiada pelo cálculo da dose equivalente humana, a qual é dez vezes maior. Nesse sentido, a dose para ser utilizada nos animais seria entre 20 mg/kg/dia e 60 mg/kg/dia por cinco dias^(21)^. Optou-se por uma subdose de 10 mg/kg/dia por cinco dias, visto a pré-condição dos animais pela isquemia-reperfusão, além de mimetizar a dose utilizada por Han e colaboradores^(16)^.

O protocolo experimental teve duração de oito dias. No primeiro dia, os animais foram anestesiados com ketamina/xilazina (75 mg/kg, 10 mg/kg; Anasedan^®^, *Vetbrands*), ambos por via intraperitoneal, e submetidos à isquemia-reperfusão (utilizando *clamps* vasculares não traumáticos) ou simulação cirúrgica de 30 minutos. Após esse período, a incisão foi fechada, e a ferida, limpa e desinfetada. Os animais foram avaliados até a recuperação anestésica e devolvidos ao biotério, onde permaneceram em repouso pelo período de 24 horas para recuperação do ato cirúrgico, sendo administrado tramadol (20 mg/kg) de oito em oito horas. Do terceiro ao sétimo dia, os animais receberam 10 mg/kg/dia por via oral de ivermectina uma vez ao dia. Após, foram colocados em gaiolas metabólicas durante 24 horas para mensuração do volume urinário, ingestão hídrica e alimentar de 24 horas. No oitavo dia, os animais foram anestesiados com ketamina/xilazina (100 mg/kg/10 mg/kg; Anasedan^®^, *Vetbrands*) por via intraperitoneal, realizando a coleta de amostra sanguínea por meio da punção da aorta abdominal. Durante o procedimento, os animais foram mantidos em superfície aquecida a 38ºC para evitar hipotermia. O rim direito foi retirado e, posteriormente, pesado para o cálculo da razão peso do rim e peso do animal. A amostra urinária foi utilizada para realização de estudos de função renal e, posteriormente, de estresse oxidativo.

Ao final do experimento, foi realizada eutanásia do animal método físico de exsanguinação, segundo as normas éticas para manuseio de animais em laboratório de pesquisa^(18)^.

### Função renal e bioquímica

Para avaliação da função renal, os níveis plasmáticos e/ou urinários de creatinina e ureia foram analisados espectrofotometricamente, de acordo com procedimentos padrão, usando *kits* diagnósticos disponíveis comercialmente (Labtest Diagnostic, MG, Brasil). A taxa de filtração glomerular foi determinada por meio de *clearance* de creatinina, calculada pela fórmula: *clearance* de creatinina = creatinina urinária x fluxo urinário (24 horas)/creatinina sérica^(22,23)^. Os níveis de ureia foram determinados com ensaio colorimétrico baseado na atividade da urease^(24)^. As proteínas urinárias foram determinadas com um método colorimétrico baseado em vermelho de pirogalol-molibdato^(25)^. Os resultados foram expressos em mg/24 horas.

### Estresse oxidativo

Os metabólitos oxidativos foram avaliados por meio de dosagem de peróxidos urinários e de substâncias reativas ao ácido tiobarbitúrico (TBARS). A avaliação de peróxidos urinários foi realizada pelo método FOX-2, em que a utilização de ferro-xilenol laranja oxida o íon Fe^2+^, produzindo um complexo de coloração azul-arroxeado (α= 4,3 x 104 M^-1^ cm^-1^)^(26-28)^. A avaliação de TBARS urinários permite a identificação de produtos finais da cascata de peroxidação lipídica, que reagem na presença do ácido tiobarbitúrico em fluídos orgânicos (α= 1,56 x 105 M^-1^ cm^-1^)^(29)^.

### Classificação da injúria renal aguda

O *Kidney Disease: Improving Global Outcomes* (KDIGO) instituiu a principal diretriz para identificação/definição da IRA e seus estágios. As classificações estão apresentadas no Quadro 1 ^(1)^.

**Quadro 1:** Classificação Kidney Disease: Improving Global Outcomes

KDIGO 1	Aumento da creatinina sérica ≥ 0,3 mg/dL (≥ 26,5 μmol/L) em até 48 horas ou aumento de 1,5 a 1,9 vezes em relação ao valor basal (ocorrido ou presumido em até sete dias), ou débito urinário < 0,5 mL/kg/hora por seis a 12 horas.
KDIGO 2	Aumento da creatinina sérica de 2,0 a 2,9 vezes em relação ao valor basal ou débito urinário menor que 0,5 mL/kg/hora por ≥ 12 horas.
KDIGO 3	Aumento da creatinina sérica de três vezes em relação ao valor basal ou creatinina sérica ≥ 4,0 mg/dL (≥ 353,6 μmol/L) ou início de terapia renal substitutiva (diálise), ou débito urinário menor que 0,3 mL/kg/hora por ≥ 24 horas ou anúria por ≥ 12 horas.

*KDIGO - Kidney Disease: Improving Global Outcomes.*

### Análise dos resultados e estatística

Os pressupostos de normalidade e homogeneidade de variâncias foram testados para todas as variáveis. Os resultados foram expressos em média ± desvio padrão quando as variáveis aderiram ao teste de normalidade. A comparação das variações entre os grupos foi comprovada por meio do teste ANOVA *One-Way*, seguido do pós-teste de comparações múltiplas de Tukey pelo programa estatístico *Graph-Pad Prism version-8* para *Windows^®^*. Foram considerados significativos os valores de p<0,05.

## RESULTADOS

Na Tabela, 1 foi possível observar que o peso do animal nos grupos Sham + ivermectina e isquemia + ivermectina variou com estatística significativa em relação aos grupos que não receberam ivermectina. Além disso, a relação peso rim/peso animal mostrou elevação no grupo isquemia + ivermectina em relação aos demais.

**Tabela 1:** Parâmetros fisiológicos dos diversos grupos, como Sham, Sham + ivermectina, isquemia, e isquemia + ivermectina, São Paulo, São Paulo, Brasil, 2024

Grupos	n	Peso do animal (g)	Peso do rim (g)	Peso rim/ peso do animal	Ingestão de ração (g)	Ingestão de água (ml)
Sham	05	292 ± 15	1,24 ± 0,09	0,42 ± 0,03	32 ± 13	38 ± 5
Sham + ivermectina	05	243 ± 28^a^	1,20 ± 0,17	0,50 ± 0,09	34 ± 5	29 ± 10
Isquemia	05	249 ± 28	1,22 ± 0,36	0,48 ± 0,10	27 ± 16	30 ± 14
Isquemia + ivermectina	05	244 ± 20 ^a^	1,80 ± 0,53	0,75 ± 0,27^a^	25 ± 14	50 ± 23

Os dados foram expressos em média ± desvio padrão;

a p<0,05 vs Sham.

Observa-se na Tabela 2 que, apesar do aumento numérico do volume urinário nos ratos medicados com ivermectina, e ainda mais nos submetidos ao processo de isquemia-reperfusão, os resultados não foram significantes. Em contrapartida, a creatinina urinária demonstrou significância no grupo isquemia + ivermectina em relação ao grupo Sham tal qual a creatinina plasmática. A ureia apresentou elevação significativa no grupo que fez a associação da ivermectina com isquemia-reperfusão. Já a análise do *clearance* de creatinina presente na Figura 1 demonstrou que os animais medicados com ivermectina apresentaram diminuição, que foi ainda maior nos grupos submetidos à isquemia-reperfusão, especialmente no grupo isquemia + ivermectina.

**Tabela 2:** Função renal dos diversos grupos, como Sham, Sham + ivermectina, isquemia, e isquemia + ivermectina, São Paulo, São Paulo, Brasil, 2024

Grupos	n	Volume urinário 24 horas (ml/min)	Creatinina urinária (mg/dL)	Creatinina plasmática (mg/dL)	Proteinúria (mg/dL)	Ureia (mg/dL)
Sham	05	0,011 ± 0,001	72,6 ± 14,5	0,30 ± 0,08	42,5 ± 5,3	39,9 ± 7,7
Sham + ivermectina	05	0,009 ± 0,002	57,9 ± 18,6	0,60 ± 0,06	44,7± 21,8	49,2 ± 18,4
Isquemia	05	0,011 ± 0,012	44,4 ± 14,1	0,69 ± 0,12	53,0 ± 14,9	61,8 ± 12,9
Isquemia + ivermectina	05	0,018 ± 0,014	27,9 ± 20,5^a^	1,26 ± 0,78^a^	42,4 ± 9,6	100,0 ± 23,2^abc^

Os dados foram expressos em média ± desvio padrão;

a p<0,05 vs Sham;

b p<0,05 vs Sham + ivermectina;

c p<0,05 vs isquemia.

**Figura 1:** *Clearance* de Creatinina dos grupos Sham (0,96); Sham + IV (0,38); Isquemia (0,35); Isquemia + IV (0,11), São Paulo, São Paulo, Brasil, 2026

*Os dados foram expressos em média ± desvio padrão. ap<0,05 vs Sham bp<0,05 vs Sham +IV cp<0,05 vs ISQ.*

As Figuras 2A e 2B mostram o perfil oxidativo. Observa-se que o tratamento com ivermectina foi responsável por aumentar os níveis de FOX-2, e quando associado ao procedimento de isquemia-reperfusão, este aumento foi ainda maior. Além disso, a observação do TBARS urinário mostra que o grupo isquemia + ivermectina apresentou aumento significativo em relação ao grupo Sham.

**Figura 2:** FOX e TBARS urinários. FOX (2A): Sham (1,74); Sham+IV (16,04); ISQ (6,98); Isq+IV (29,66). TBARS (2B): Sham (16,19); Sham+IV (20,40); ISQ (25,93); Isq+IV (32,01), São Paulo, São Paulo, Brasil, 2026

*Os dados foram expressos em média ± desvio padrão. ap<0,05 vs Sham bp<0,05 vs Sham +IV cp<0,05 vs ISQ.*

## DISCUSSÃO

O presente estudo investigou a relação entre a administração de ivermectina e a ocorrência de injúria renal, em um modelo experimental de isquemia-reperfusão em ratos Wistar, ao longo de cinco dias. Observou-se redução da função renal associada a intenso estresse oxidativo tanto nos animais que receberam ivermectina isoladamente quanto naqueles submetidos ao fator de risco isquemia-reperfusão. Esses achados corroboram a hipótese deste estudo quanto ao potencial efeito nefrotóxico da ivermectina.

O grupo dos animais que recebeu ivermectina apresentou variação de peso significativa durante a realização do protocolo experimental. O processo inflamatório motivado pela ingestão da ivermectina e o procedimento cirúrgico de isquemia-reperfusão, que resulta em consumo de energia por parte das células, foram os principais precursores para esse fenômeno de redução do peso. A liberação de citocinas pró-inflamatórias afeta nervos periféricos direta ou indiretamente, que estimulam áreas do cérebro induzindo anorexia, na tentativa de priorizar energia para reparação de tecidos e estruturas, mitigando a presença de agentes patógenos ou tóxicos^(30,31)^.

Embora a nefrotoxicidade da ivermectina ainda não esteja completamente esclarecida, estudo pré-clínico demonstrou redução da função renal e lesão no parênquima renal em animais tratados por quatro dias com 40 mg/kg de ivermectina - dose equivalente à administrada em seres humanos^(32,33)^. O presente estudo mostrou elevação dos biomarcadores renais após o uso de 10 mg/kg de ivermectina por cinco dias, corroborando os dados anteriores e reforçando a hipótese de toxicidade renal. Essa toxicidade pode ser mediada por dano tubular direto, envolvendo alterações na polaridade das membranas celulares, com deslocamento das proteínas da bomba Na^+^/K^+^-ATPase da membrana basolateral para a apical, desencadeando apoptose celular e lesão no DNA^(34-36)^.

Nesse contexto, estudo experimental que administrou 12 mg/kg de ivermectina em ratos Wistar por cinco dias demonstrou redução da atividade da Na^+^/K^+^-ATPase no córtex cerebral, sugerindo uma possível correlação do fármaco com a desregulação da homeostase celular^(37)^.

Dessa forma, no parênquima renal, pode-se observar um desequilíbrio nos níveis de água, sódio, potássio e bicarbonato, além da elevação de biomarcadores como ureia e creatinina plasmática, o que pode culminar em quadros de oligúria ou poliúria^(4,5)^.

No presente estudo, os animais que foram submetidos à isquemia e receberam a ivermectina apresentaram uma elevação do volume urinário. Esse fenômeno pode ocorrer em modelos de IRA isquêmica, visto que a hipóxia persistente pode causar vacuolização das células dos túbulos proximais, resultante do acúmulo de lisossomos gigantes, os quais também são frequentes nas IRAs por fármacos^(34,35)^. Assim, apresenta-se inesperado aumento do volume urinário pelo mecanismo conhecido como nefrose osmótica.

Em uma visão translacional, a vigilância e avaliação do volume urinário em pacientes de risco para IRA, seja na comunidade ou no ambiente especializado de cuidados hospitalares, podem ser mitigadas em detrimento de mecanismos fisiopatológicos específicos, como o relatado acima. Logo, não só a avaliação do volume urinário é fundamental, mas também a análise de biomarcadores renais como ureia e creatinina, bem como a avaliação clínica por meio de sinais e sintomas como edema e congestão pulmonar.

Na tentativa de racionalizar e intervir precocemente na vigilância clínica da IRA, o KDIGO vem se consolidando como consenso entre os principais nefrologistas do mundo^(1)^. Essa ferramenta estabelece critérios para identificação e estratificação da IRA, orientando a equipe multiprofissional no diagnóstico precoce com o objetivo de promover uma intervenção rápida e favorecer a recuperação do paciente.

Neste cenário, o presente estudo demonstrou, por meio do *clearance* de creatinina e dos níveis de creatinina sérica, que os grupos experimentais pré-clínicos podem ser associados aos diferentes estágios propostos pelo KDIGO. Assim, em uma transposição da bancada para prática clínica, os animais do grupo controle poderiam corresponder a pacientes saudáveis (KDIGO 0); os grupos que receberam ivermectina, a pacientes com desenvolvimento de IRA nefrotóxica isolada (KDIGO 1); o grupo submetido à isquemia, ao estágio KDIGO 2; e o grupo isquemia + ivermectina, ao estágio KDIGO 3^(1,2)^.

Com o objetivo de reforçar a hipótese da IRA induzida pela ivermectina, os metabólitos oxidativos avaliados neste estudo demonstraram participação significativa de EROs, especialmente nos grupos tratados com ivermectina e nos submetidos à IRA isquêmica.

A análise do TBARS urinário permite o monitoramento da peroxidação lipídica, processo que envolve a degradação de compostos lipídicos pela ação de agentes oxidantes, resultando em alterações na permeabilidade das membranas celulares, formação de compostos tóxicos e, consequentemente, morte celular^(21)^. Já a análise do método FOX-2 possibilita a detecção de níveis de peróxidos, cuja elevação está associada à intensificação da produção de EROs decorrentes do estresse oxidativo^(21,22)^.

Os dados do presente estudo demonstraram aumento significativo nos níveis de TBARS urinário e FOX-2, especialmente nos grupos submetidos à isquemia e isquemia associada à administração de ivermectina. Esse achado pode ser explicado, principalmente, pelos mecanismos envolvidos no processo de isquemia-reperfusão, uma vez que a reperfusão promove um aumento abrupto da concentração de oxigênio, ativando células endoteliais e recrutando mediadores inflamatórios e células do sistema imune, o que intensifica o estresse oxidativo^(38)^. Além disso, o grupo controle tratado com ivermectina apresentou um aumento estatisticamente significativo nos níveis de FOX-2, sugerindo que a ivermectina pode atuar como um composto pró-oxidante, favorecendo a formação de peróxidos.

Estudo recente com ratos Wistar avaliou a neurotoxicidade da ivermectina, demonstrando aumento dos níveis de creatinina sérica nos animais que receberam, durante cinco dias, uma dose de 12 mg/kg do fármaco^(37)^. Além disso, foi observada uma elevação em EROs e uma redução na atividade da enzima antioxidante catalase. Outro estudo sobre o uso crônico da ivermectina, na dose de 0,4 mg/kg, durante quatro semanas, revelou redução das atividades das enzimas antioxidantes, piora nos biomarcadores renais, alterações histológicas e aumento da expressão da proteína Bax de caráter pró-apoptótico^(39)^.

A hipóxia persistente no córtex renal favorece a produção exacerbada de EROs, o que compromete a fosforilação oxidativa mitocondrial, além de causar depleção de óxido nítrico, formação de padrões moleculares associados a dano (DAMPs), ativação de receptores Toll-like (TLRs), indução de autofagia e disfunção microvascular^(36,38)^.

Nesse contexto, os DAMPs liberados por células tubulares renais necróticas ativam os TLRs presentes nas células renais, desencadeando uma cascata que envolve a ativação do fator de transcrição NF-κB. Esse processo resulta na translocação nuclear do NF-κB, promovendo a expressão de citocinas inflamatórias como fator de necrose tumoral e interleucina-6, retroalimentando o processo de lesão renal^(36,38)^.

Portanto, o presente estudo buscou elucidar os mecanismos fisiopatológicos envolvidos na toxicidade renal induzida pela ivermectina por meio de avaliação da função renal e do desequilíbrio redox no modelo experimental em ratos Wistar. Os dados apresentados poderão contribuir para construção de novos paradigmas sobre o uso desse fármaco, especialmente em pacientes com fatores de risco.

### Limitações do estudo

Este estudo apresenta como limitação a ausência de dados histológicos que permitam demonstrar as alterações morfológicas renais associadas ao uso da ivermectina. Além disso, não foi realizada uma análise da relação dose-resposta em modelos experimentais com fatores de risco, apesar de a literatura já ter descrito o uso de doses (19 mg/kg) consideradas seguras do fármaco em ratos Wistar^(39)^.

### Contribuições para as áreas da enfermagem, saúde ou políticas públicas

A enfermagem, enquanto referência no cuidado integral ao paciente, deve conhecer não apenas os efeitos das medicações administradas, mas também suas interações com potenciais fatores de risco. Esse conhecimento é essencial para adoção das melhores práticas no atendimento a pacientes com IRA.

## CONCLUSÕES

A ivermectina é um medicamento potencialmente nefrotóxico, e seu uso deve ser cauteloso, pois esta foi capaz de alterar biomarcadores renais, indicando piora da função renal, e ativar o processo oxidativo. Dessa forma, neste estudo experimental em ratos Wistar, a IRA por isquemia-reperfusão se caracterizou como fator de risco para a nefrotoxicidade da ivermectina.

## Data Availability

Os dados de pesquisa estão disponíveis no corpo do artigo.
